# EMF Antenna Exposure on a Multilayer Human Head Simulation for Alzheimer Disease Treatments

**DOI:** 10.4236/jbise.2022.155013

**Published:** 2022-05-25

**Authors:** Felipe P. Perez, Maryam Rahmani, John Emberson, Makenzie Weber, Jorge Morisaki, Farhan Amran, Syazwani Bakri, Akmal Halim, Alston Dsouza, Nurafifi Mohd Yusuff, Amran Farhan, James Maulucci, Maher Rizkalla

**Affiliations:** 1 Department of Medicine, Division of General Internal Medicine and Geriatrics, Indiana University School of Medicine, Indianapolis, IN, USA;; 2 Department of Electrical and Computer Engineering, Indiana University-Purdue University, Indianapolis, IN, USA;; 3 Department of Bioengineering, University of Illinois at Chicago, Chicago, IL, USA

**Keywords:** EM, Antenna, Brain Tissues, Simulation, Alzheimer

## Abstract

In this paper, we follow up with our preliminary biological studies that showed that Repeated electromagnetic field stimulation (REMFS) decreased the toxic amyloid-beta (A*β*) levels, which is considered to be the cause of Alzheimer’s disease (AD). The REMFS parameters of these exposures were a frequency of 64 MHz and a Specific absorption rate (SAR) of 0.4 to 0.9 W/Kg in primary human neuronal cultures. In this work, an electromagnetic field (EMF) model was simulated using high-frequency simulation system (HFSS/EMPro) software. Our goal was to achieve the EM parameters (EMF Frequency and SAR) required to decrease the toxic A*β* levels in our biological studies in a simulated human head. The simulations performed here will potentially lead to the successful development of an exposure system to treat Alzheimer’s disease patients. A popular VFH (very high frequency) patch microstrip antenna system was considered in the study. The selection was based on simple and easy construction and appropriateness to the VHF applications. The evaluation of the SAR and temperature distribution on the various head layers, including skin, fat, dura, the cerebrospinal (CSF), and grey matter, brain tissues, were determined for efficacy SAR and safety temperature increase on a simulated human head. Based on a current pulse of 1 A peak current fed to the antenna feeder, a maximum SAR of 0.6 W/Kg was achieved. A range of 0.4 to 0.6 SAR was observed over the various layers of the simulated human head. The initial design of the antenna indicated an antenna size in the order of 1 m in length and width, suggesting a stationary practical model for AD therapy. Future direction is given for wearable antenna and exposure system, featuring high efficiency and patient comfort.

## 1. INTRODUCTION

Alzheimer’s disease (AD) had been spreading worldwide with little development in treatment. The number of people living with Alzheimer’s disease (AD) and other dementias worldwide was estimated at 46 million in 2015, with an estimated prevalence reaching 131 million in 2050 [1]. Research efforts to slow down, or stop the disease, will potentially impact the healthcare system and the global economy. AD is a complex and heterogeneous disorder. The drugs approved by the U.S. Food and Drug Administration (FDA) for the treatment of AD do not stop the progression of the disease [2]. Research efforts have also examined strategies with antibody treatment against A*β* and alternative modalities to slow down the disease progress [3, 4]. Given the poor tolerability of pharmaceuticals in the treatment of AD, there is a growing interest to explore other potential noninvasive mechanisms including EMF exposures. A recent clinical trial of electromagnetic exposure (915 MHz) to AD patients found no significant side effects or physiologic changes [5]. Active research in the use of noninvasive brain stimulation as a potential therapy for AD has included several pilot studies and small clinical trials that have highlighted the potential for neuroenhancement and improvement in cognitive function in healthy individuals via noninvasive brain stimulation (NBS) [6]. Mechanism of action, efficacy, and reproducibility [7–9] were issues to be further addressed in future research. The EMF clinical trial mentioned above used 915 MHz with a high frequency is probably not effective in the sense that radiation does not reach important deep tissues of a human head (only 3 to 4 cm tissue penetration depth). In our proposed efforts in this study, we explore the use of repeated electromagnetic field stimulation (REFMS), as a non-invasive potential strategy to lower the A*β* peptide load observed in AD. REMFS uses a non-thermal mechanism to produce biological effects, this occurs at the molecular level and involves multitarget interactions between signaling pathways [10, 11]. Computer simulations for EMF exposures at a frequency of 64 MHz and a SAR of 0.4 – 0.9 W/Kg may establish a framework for safe human exposure [12–14]. Overall efforts to develop noninvasive therapeutic strategies point to the development of portable devices for AD treatment.

In this research work, the repeated electromagnetic field stimulation (REMFS) was found to be decreasing the toxic amyloid-beta (A*β*) levels [15], which is considered to be the cause of Alzheimer’s disease (AD). HFSS was utilized to obtain the antenna parameters and the field distribution following the preliminary results reported in [15].

## 2. THE FIELD MODEL

The following field equations that are combined to determine the field distribution and the SAR values are given by Maxwell’s equations combined with the heat equations over the various boundaries of the human head tissues. The solutions of the following equations via HFFS/EMPro give the field and SAR distribution over the human head tissues.

(1) ∇×E=jωμH

(2) ∇×H=−jωεE

(3) ∇⋅E=0

(4) ∇⋅H=0

where ***E*** is the electric field, ***H*** is the magnetic field, *ω* is the radian frequency, *μ* is the mobility, and *ε* is the permittivity of the material.

With the lossy media, considering the conductivity, *σ*, the power equation is given as [16, 17]:

(5) $$P_{v}=2\pi f\varepsilon_{0}\varepsilon \prime \prime E^{2}$$

The average power as function of the electric field, *E*, in lossy media is given by:

(6) $$P_{av}=\int 0.5\sigma \left|E^{2}\right|\text{d}v$$

where d*v* is the differential volume.

The dielectric loss in a material due to electric conductivity is given by:

(7) $$\varepsilon \prime \prime =\sigma /2\pi \varepsilon_{0}f$$

The microwave dissipated power term is considered as the source term in Fourier heat transfer equation [18]:

(8) $$\rho C_{p}\partial T/\partial t=k\nabla^{2}T+P_{v}(x,y,z,t)$$

where *ρ* is the mass density, and *C*_*p*_ is the specific heat capacity.

The SAR may be evaluated by the equation:

(9) $$\text{SAR}=(\sigma /2\rho )E^{2}$$

The change in thermal energy/temperature change is given by:

(10) $$q=mC_{p}\nabla T$$

The SAR is then can be calculated by ΔSAR = *C*_*p*_Δ*T/*Δ*t*, where Δ*T* is the change in temperature related to change in SAR by a value of ΔSAR over time Δ*t*.

## 3. ANTENNA STRUCTURE

Based on the 64 MHz radiation pattern, the dimensions of the patch antenna are given in the range of 1.07 m × 1.38 m. The Matlab estimation is based on the following equations:

The width of the patch, *w*, is given by [19]:

(11) $$W=\frac{v_{0}}{2f_{r}}\sqrt{\frac{2}{\varepsilon_{\text{reff}}+1}}$$

where *v*_0_ = speed of light, and *f_r_* is the desired frequency. The effective dielectric constant is given by [18]:

(12) $$\varepsilon_{r,eff}=\frac{\varepsilon_{r}+1}{2}+\frac{\varepsilon_{r}-1}{2}{\left(1+12\frac{h}{W}\right)}^{-0.5}$$

where *ε*_*r*_ = dielectric constant of the substrate; *ε*_*r,eff*_ = effective dielectric constant: 1< ε_*r,eff*_ < *ε*_*r*_.

The extension length, Δ*L*, is given by:

(13) $$\Delta L=0.412h\frac{\left(\varepsilon_{r,eff\;}+0.3\right)\left(\frac{W}{h}+0.264\right)}{\left(\varepsilon_{r,eff\;}-0.258\right)\left(\frac{W}{h}+0.8\right)}$$

where *h* = height of the dielectric substrate.

The length of the patch, *L*, and the notch width, *g*, are given by:

(14) $$L=\frac{v_{0}}{2f_{r}\sqrt{\varepsilon_{r,eff\;}}}-2\Delta L$$

(15) $$g=\frac{v_{0}}{\sqrt{2\varepsilon_{eff}}}\frac{4.65\times 10^{-12}}{f}$$

The inset feed depth, *Fi* is given by:

(16) $$Fi=10^{-4}(0.0016922\varepsilon_{r}^{7}+0.13761\varepsilon_{r}^{6}-6.1783\varepsilon_{r}^{5}+93.187\varepsilon_{r}^{4}-682.69\varepsilon_{r}^{3}+2561.9\varepsilon_{r}^{2}-4043\varepsilon_{r}+6697)\frac{L}{2}$$

The feed line width, *W*_*f*_, is given by:

(17) $$\frac{W_{f}}{2}=\{\begin{array}{ll} \frac{8{\text{e}}^{A}}{{\text{e}}^{2A}-2}, & \frac{W_{f}}{2}<2\\ \frac{2}{\pi }\left(B-1-\text{ln}(2B-1)+\frac{\varepsilon_{r}-1}{2\varepsilon_{r}}\left[\begin{array}{c} \text{ln}(B-1)+0.39\\ -\frac{0.61}{\varepsilon_{r}} \end{array}\right]\right), & \frac{W_{f}}{2}>2, \end{array}$$

where *A* & *B* are defined as:

(18) $$A=\frac{Z_{0}}{60}\sqrt{\frac{\varepsilon_{r}+1}{2}+\frac{\varepsilon_{r}+1}{\varepsilon_{r-1}}\left(0.23+\frac{0.11}{\varepsilon_{r}}\right)}$$

(19) $$B=\frac{377\pi }{2Z_{0}\sqrt{\varepsilon_{r}}}$$

where *Z*_0_ = characteristic impedance, and *Z*_*in*_ = input impedance.

The data given are: *h* = 0.01, *f*_*r*_ = 64 MHz, *Z*_0_ = 50Ω, and *ε*_*r*_ = 4.8.

## 4. SIMULATION RESULTS

**Figure 1.**
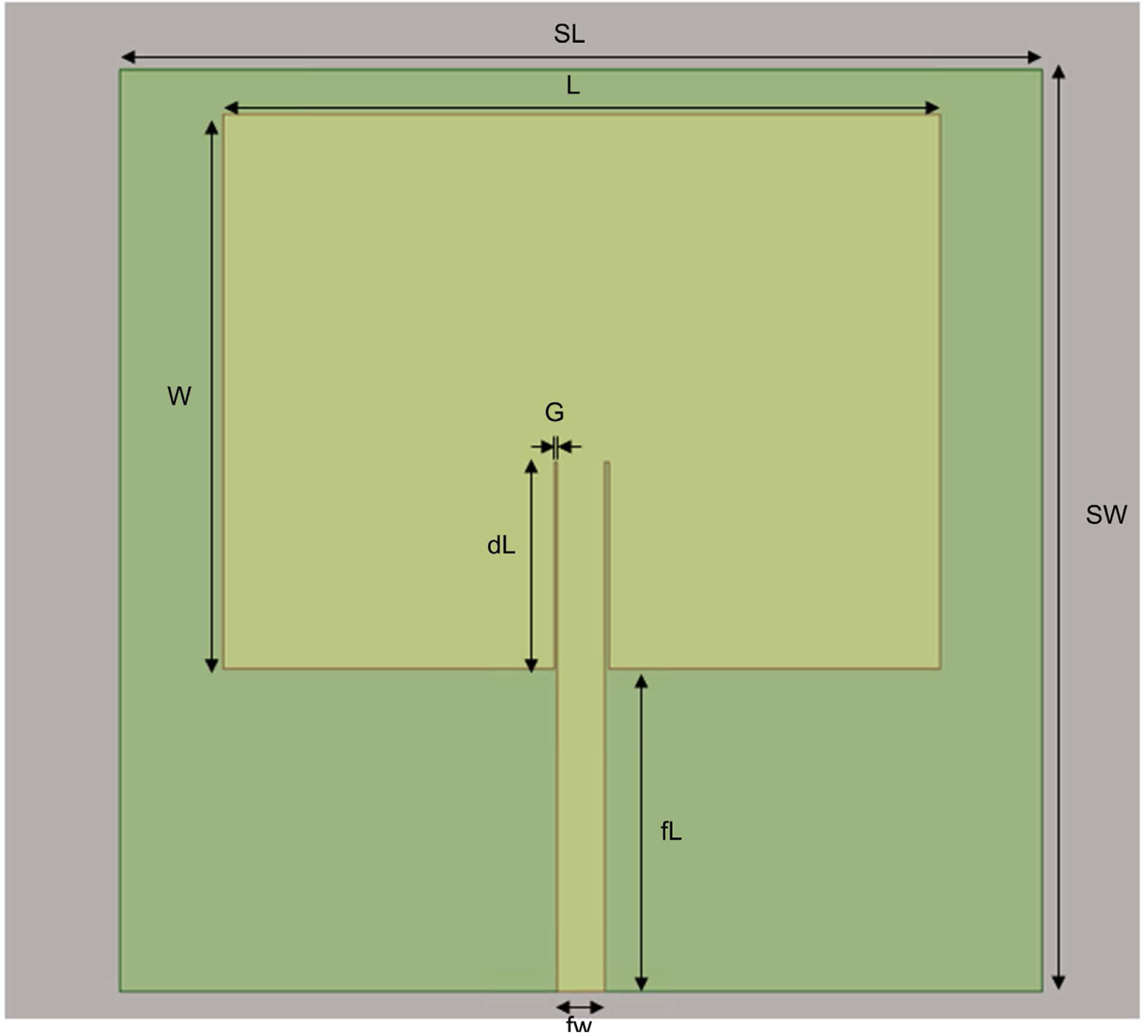

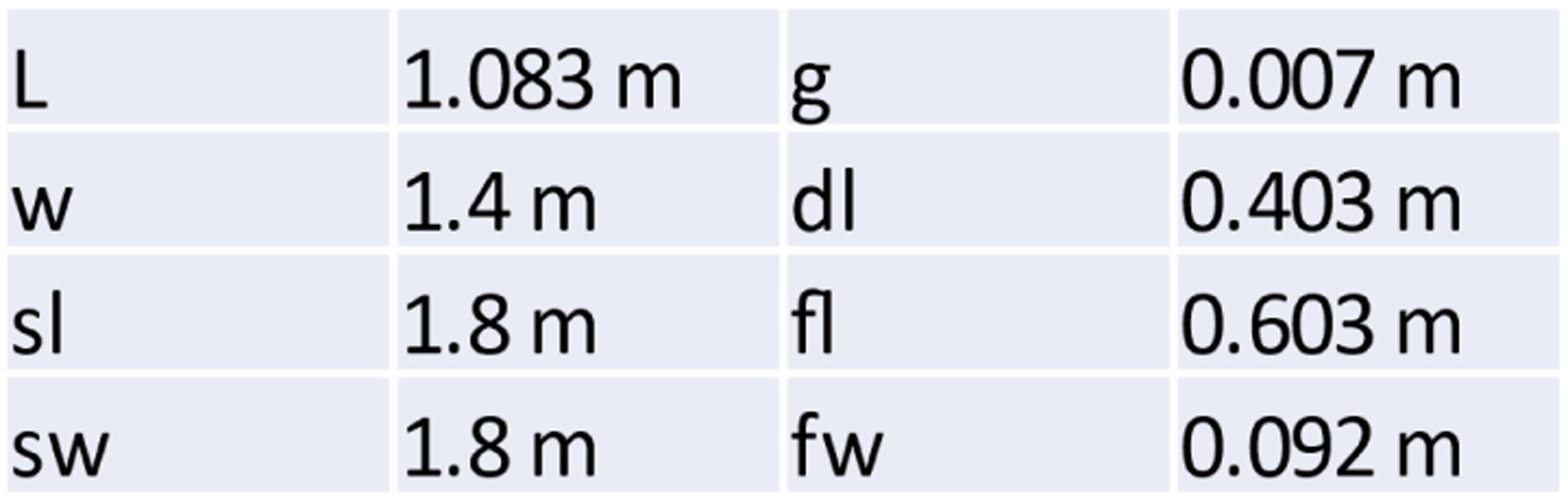
The patch antenna structure.

**Figure 2.**
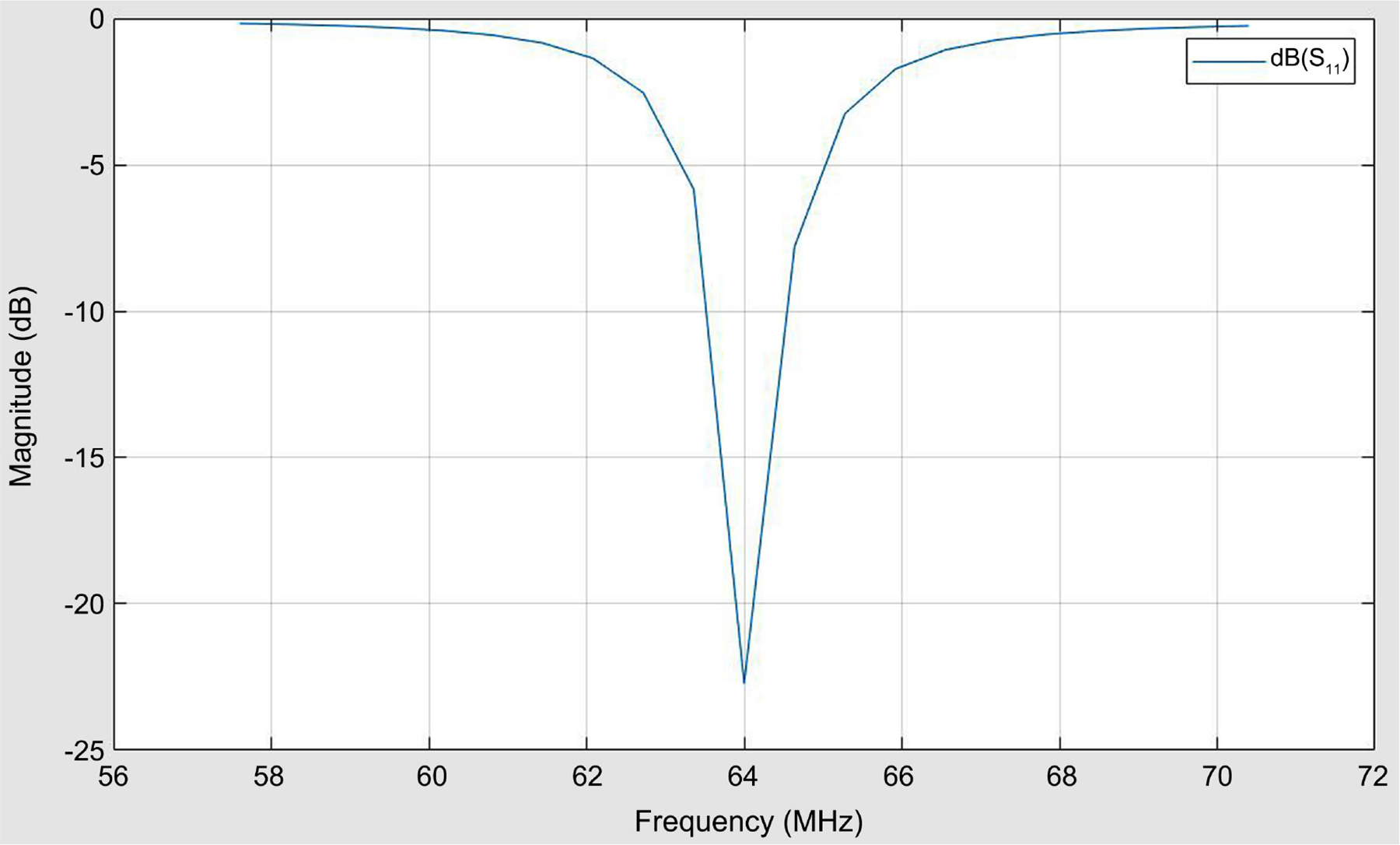
S_11_ parameter indicating radiation at 64 MHZ with −20 dbs magnitude.

**Figure 3.**
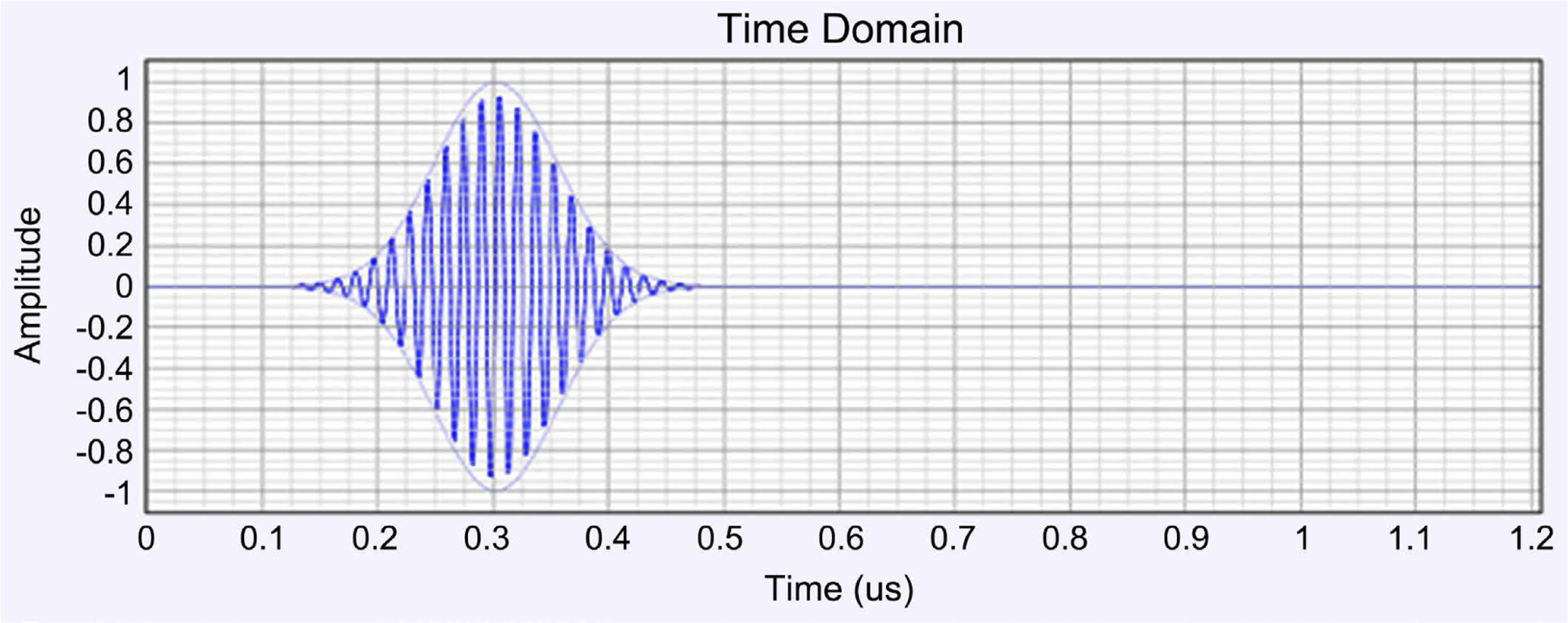
The current pulse applied to the antenna feed to produce the 0.6 SAR required SAR.

HFSS/EMPro was used in the estimation of the scattering S_11_ parameter, the Antenna SAR distribution with and without brain tissues, the E field, and SAR at the various layers of the simulated human head. The antenna device was estimated for a 64 MHz homogeneous pattern and 0.6 SAR in the brain simulation. Figure 1 gives the patch antenna structure. The scattering S_11_ parameter is given in Figure 2, and the current pulse that fed the antenna is given in Figure 3.

**Figure 4.**
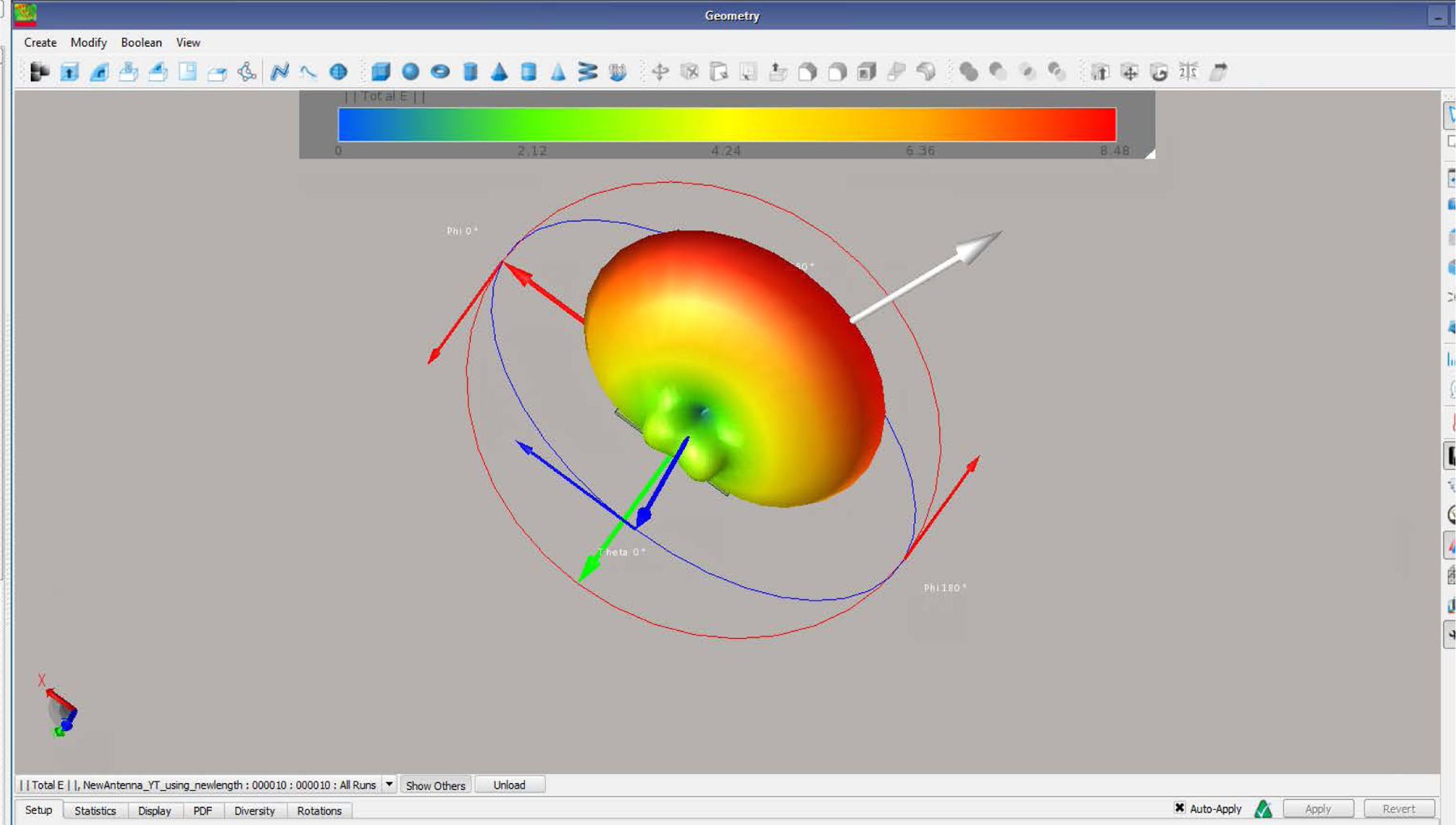
The *E* field distribution from the feed region at *x* = 0 till *E*_max_ of 8.5 V/m.

**Figure 5.**
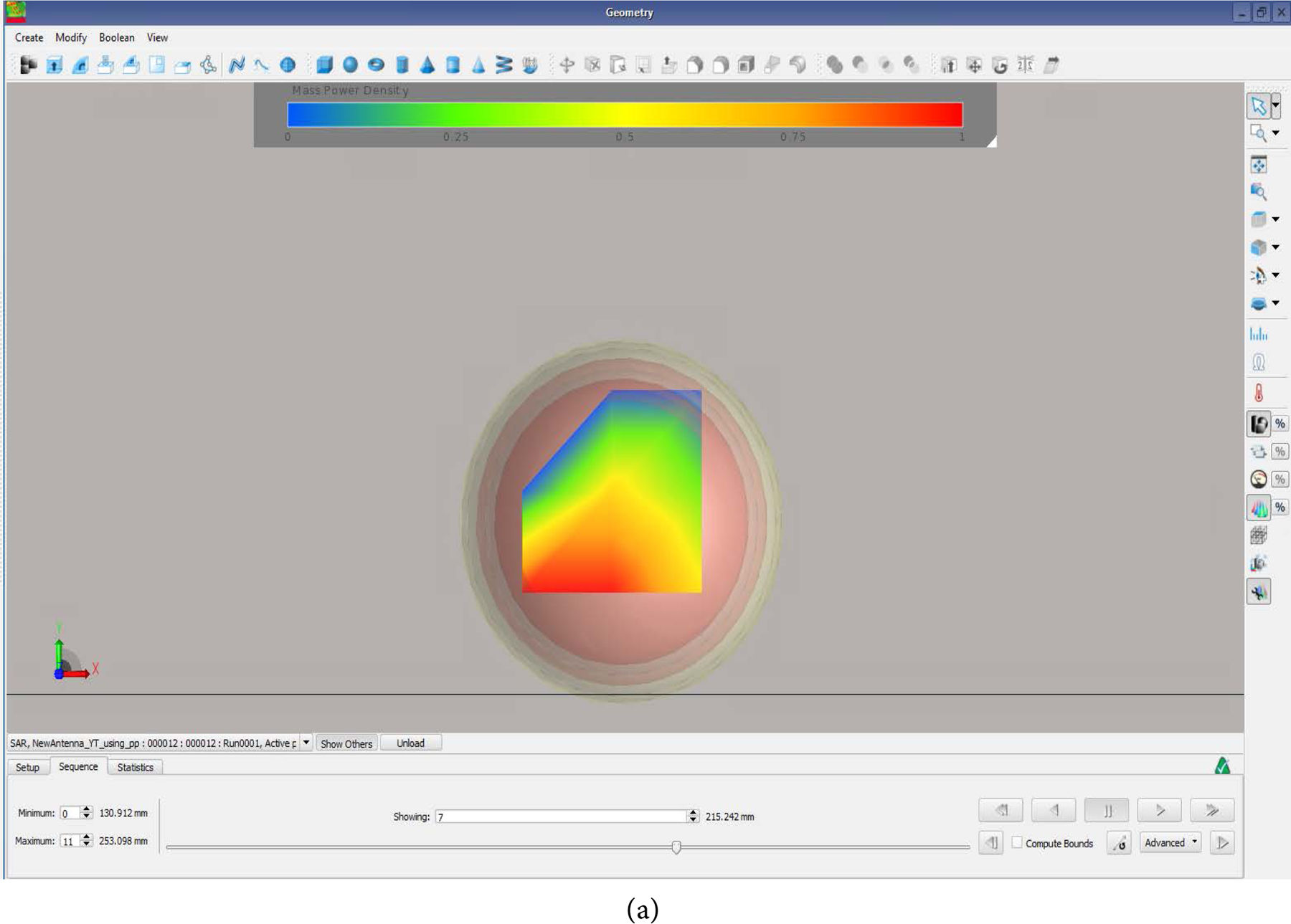

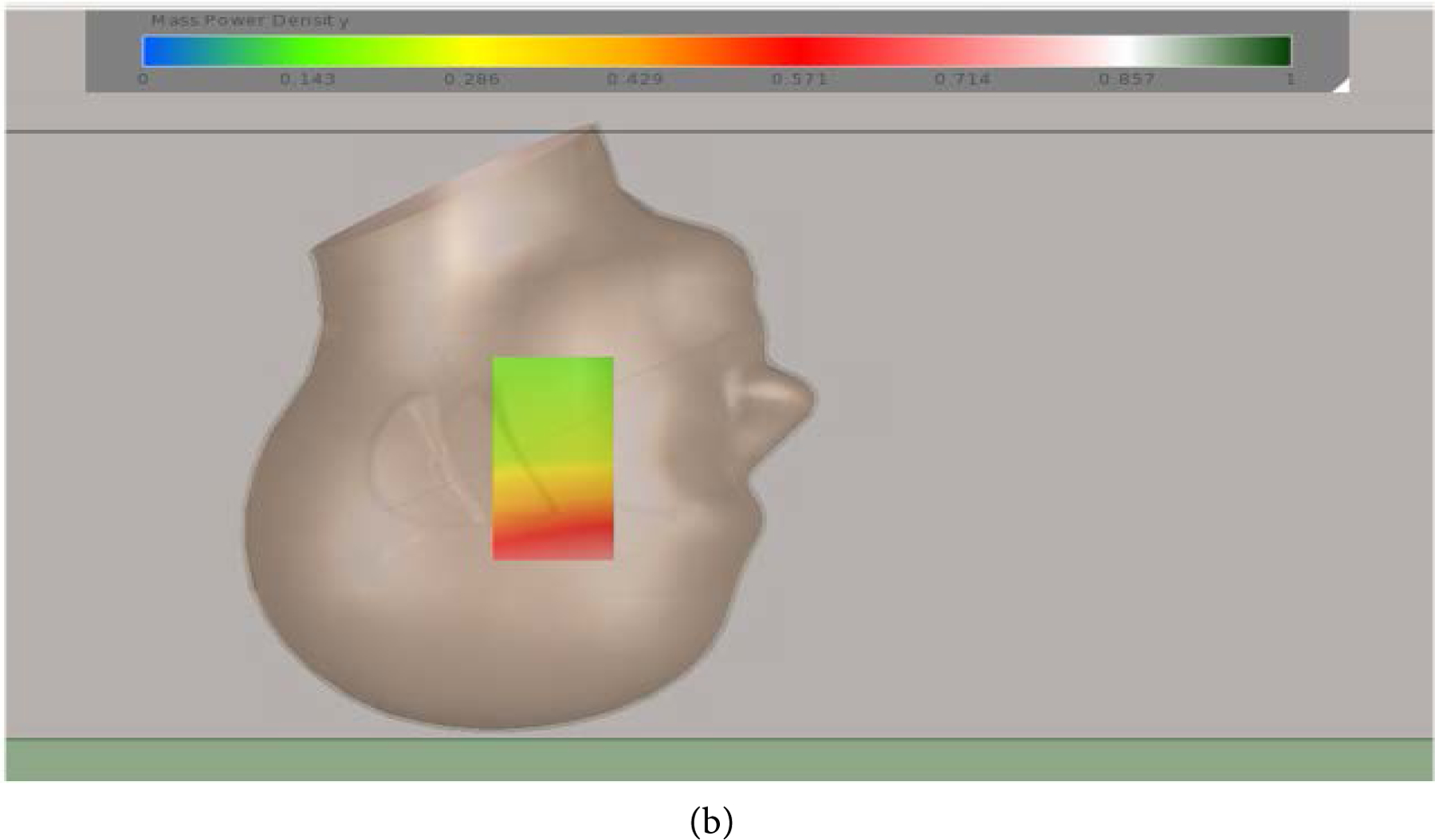
The SAR distribution: (a) in the XY plane, and (b) in the XZ plane. SAR values within tissues 0.2 to 0.9 SAR.

**Figure 6.**
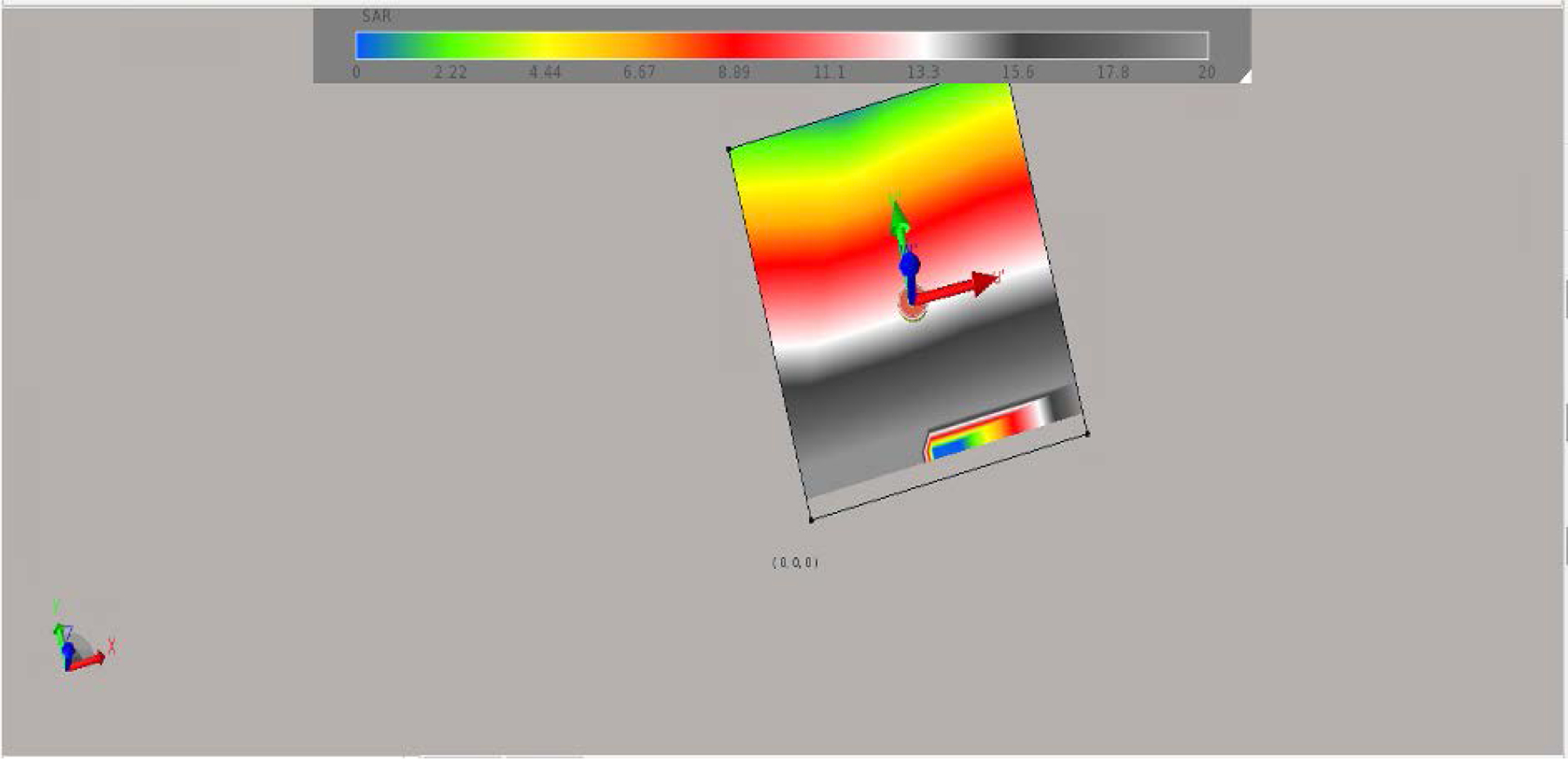
The SAR value generated by the antenna before application in the simulated brain tissue.

The antenna E-field is given in Figure 4. With maximum field given in the x-direction reaching the simulated brain. The SAR distribution in the XY plane and the XZ plane are given in Figure 5(a) and Figure 5(b) respectively. As it can be seen the SAR ranges from 0.2 to 0.9 in the simulated brain tissue. The SAR value decreased as the power radiated decreased from the brain tissue out towards the skin. The SAR value was higher before the power was absorbed by the human head. Figure 6 shows that the simulated antenna provides SAR values that range from 2 to 9 W/kg before simulated human head absorbs the EMF energy.

The 64 MHZ radiating frequency was appropriate for the penetration depth needed to reach multiple layers until the brain tissue. With enough penetration depth, the current need to drive the antenna and to reach the required power is reasonably well. As it can be seen, the input power is sufficient to reach the required SAR. The 64 HMz is also appropriate for the medical applications since it is the same frequency used for the MRI imaging. The data with the big size patch antenna suggests a stationary practical model for the EMP device Alzheimer treatment. The matching impedance from the feeder to the antenna should be considered in order to optimize the power transmission into the head phantom.

## 5. CONCLUSION AND FUTURE WORK

**Figure 7.**
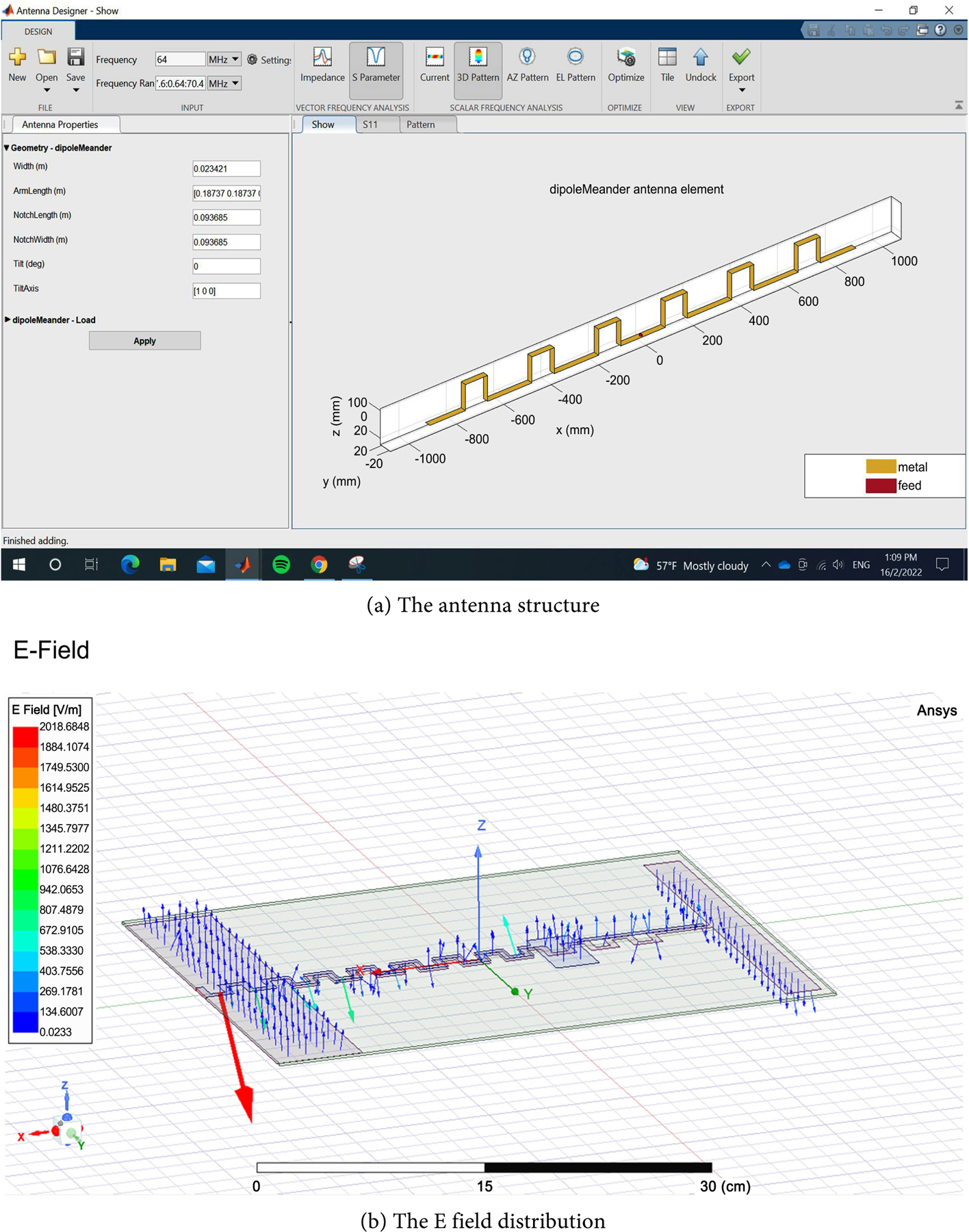
Future proposed antenna for wearable device system, (a) the antenna structure, and (b) the field distribution.

In this work, we successfully used HFSS in estimating the SAR values at the various tissues of a simulated human head with a simple strip antenna. The size of the antenna was found to be in the meter range, suggesting a stationary EMR system to be used for AD therapy. A meander line antenna [20] may be a potential candidate for miniaturizing an antenna system in order to achieve a wearable comfortable system with manageable wearable devices. Figure 7 gives a proposed antenna for wearable device system in the helmet form. The system was initially simulated and the field distribution was given in Figure 7(b). The details of this antenna wearable system are reserved for future considerations.

**Figure 8.**
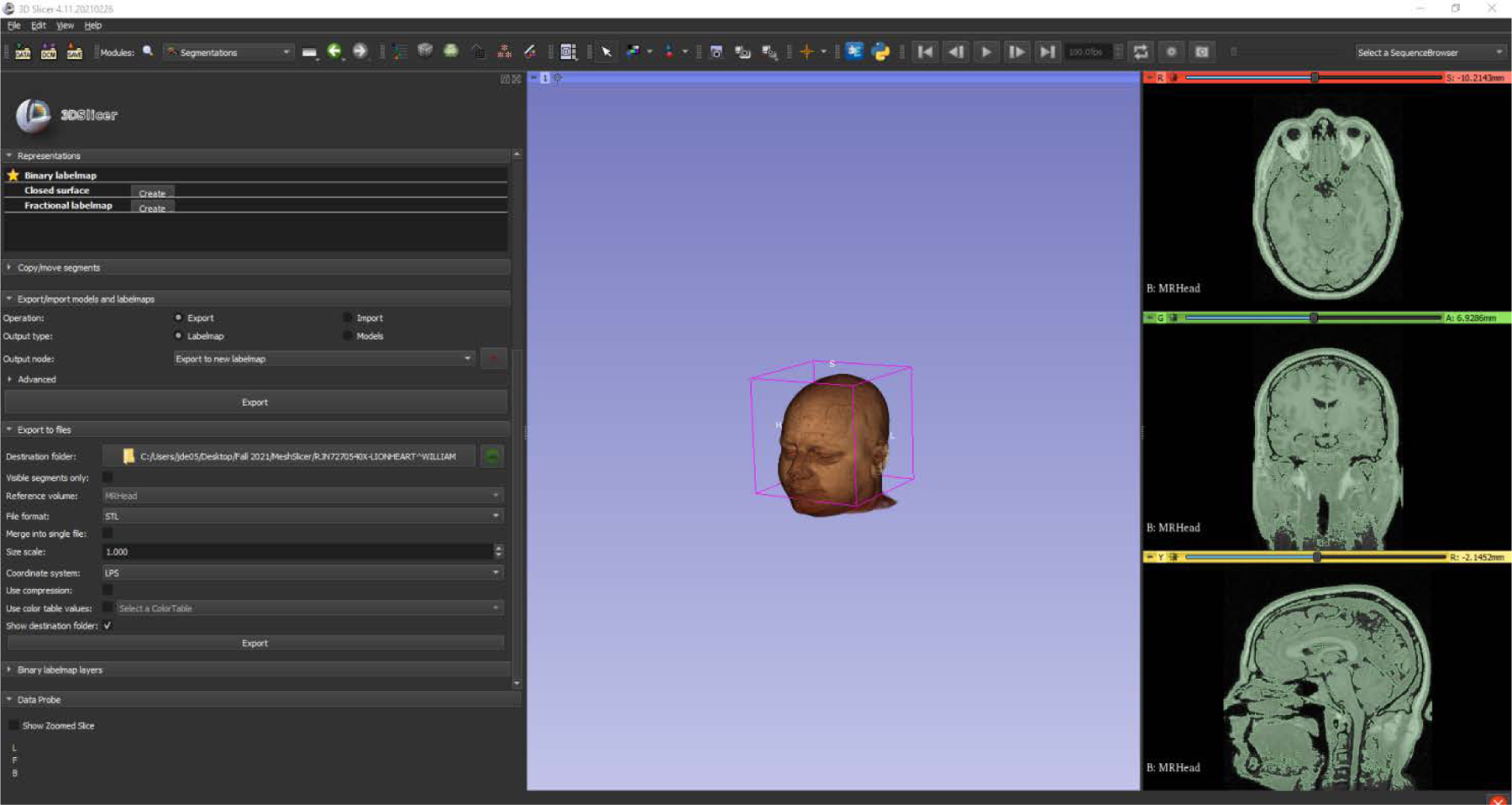
Proposed head phantom simulation with detailed anatomic layers for enhancing SAR results.

An accurate human model is essential in wearable antenna design. The accuracy of the simulation may be enhanced by the *Zubal* anthropomorphic phantom where original X-ray CT *images* were reconstructed [21]. Figure 8 shows the head phantom under consideration for future work. Also, the accuracy of the simulation for individual patients can be enhanced using Digital Imaging and Communication in Medicine (DICOM) based anthropomorphic phantom voxel data [22].
